# Thin-film electroencephalographic electrodes using multi-walled carbon nanotubes are effective for neurosurgery

**DOI:** 10.1186/1475-925X-13-166

**Published:** 2014-12-15

**Authors:** Kousuke Awara, Ryuhei Kitai, Makoto Isozaki, Hiroyuki Neishi, Kenichiro Kikuta, Naoki Fushisato, Akira Kawamoto

**Affiliations:** Department of Neurosurgery, University of Fukui, 23 Shimoaizuki Eiheijimatsuoka, Yoshida-gun, Fukui, 910-1193 Japan; Electrical and Electronic Engineering, Fukui National College of Technology, Shimoji, Sabae-city, Fukui, 916-8507 Japan

**Keywords:** Carbon nanotube, Electroencephalography, Intraoperative monitoring

## Abstract

**Background:**

Intraoperative morphological and functional monitoring is essential for safe neurosurgery. Functional monitoring is based on electroencephalography (EEG), which uses silver electrodes. However, these electrodes generate metal artifacts as silver blocks X-rays, creating white radial lines on computed tomography (CT) images during surgery. Thick electrodes interfere with surgical procedures. Thus, thinner and lighter electrodes are ideal for intraoperative use.

**Methods:**

The authors developed thin brain electrodes using carbon nanotubes that were formed into thin sheets and connected to electrical wires.

**Results:**

The nanotube sheets were soft and fitted the curve of the head very well. When attached to the head using paste, the impedance of the newly developed electrodes was 5 kΩ or lower, which was similar to that of conventional metal electrodes. These electrodes can be used in combination with intraoperative CT, magnetic resonance imaging (MRI), or cerebral angiography. Somatosensory-evoked potentials, auditory brainstem responses, and visually evoked potentials were clearly identified in ten volunteers. The electrodes, without any artifacts that distort images, did not interfere with X-rays, CT, or MR images. They also did not cause skin damage.

**Conclusions:**

Carbon nanotube electrodes may be ideal for neurosurgery.

## Background

Modern neurosurgical treatment requires continuous electrophysiological monitoring, in addition to intraoperative CT, MRI, and cerebral angiography [1–4]. Functional monitoring is based on electroencephalography (EEG), and conventional EEG electrodes are made of silver (Ag) and silver chloride (AgCl) [5–7]. However, these electrodes generate metal artifacts as silver blocks X-rays, creating white radial lines on CT images during surgery, which may hinder surgeons from identifying the underlying tissues. Thick electrodes sometimes interfere with surgical procedures, and, when disinfectants or other fluids, including blood and water, are present on the scalp, heavy electrodes may easily become detached. Electrodes easily become contaminated with blood and body fluids as they are used in the vicinity of a surgical field. Therefore, electrodes should not be re-used from the viewpoint of the prevention of infectious diseases. In recent years, carbon nanotubes have attracted attention for their notable mechanical strength and electrical characteristics [8]. The electrical current density in a multi-walled carbon nanotube is 1,000 times higher than that in a copper one [9]. To use simultaneously with MRI, CT or X-ray, particularly in a neurosurgical operating room, the specification for brand-new electrodes should be lighter, thinner, non-metallic, radiolucent and stable for liquid sterilization. We have developed a new electrode using multi-walled carbon nanotubes and examined its usability.

## Methods

### Multi-walled carbon nanotube electrodes

**Figure 1:**
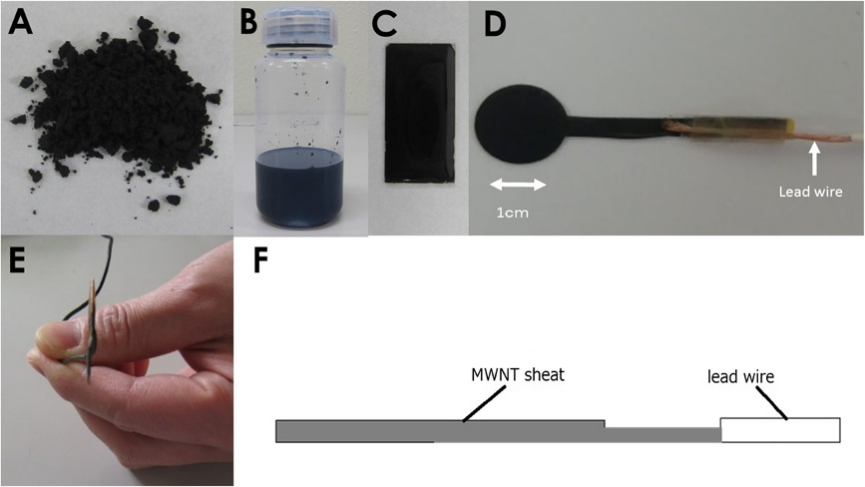
**Manufacturing process of MWCNT dispersed electrode. A**: MWCNT powder. **B**: MWCNT dispersed with PMMA. **C**: MWCNT containing solution was fixed on glass plate. **D**: Frontal view of round electrodes connected to conventional electrical wires. **E**: Lateral view of paper-like, ultra-thin electrodes. The thickness was 250 μm. **F**: Diagram.

Multi-walled carbon nanotubes (MWCNT) with a diameter of 140 nm and a length of 7 μm (Materials and Electrochemical Research Corporation, AZ USA) were used for the newly-developed electrode (Figure 1A). A mixture of MWCNT powder and poly-methyl-methacrylate (PMMA: Nacalai Tesque, Kyoto, Japan) was dispersed in Methyl Ethyl Ketone solution. The solution was exposed to ultrasonic waves for 40 minutes, using a 600-Watt homogenizer and was placed overnight (Figure 1B). After the organic solvent had vaporized over 24 hours, a thin film was formed on the glass substrate (Figure 1C). The MWCNT-containing film was shaped the same as a silver electrode with a diameter of 1.0 cm [10]. The MWCNT electrode was then connected to commercially available EEG electrodes with varying concentrations (Figure 1 D) (NE-132B Nihonkoden, Tokyo, Japan). To clarify the fundamental physical specification, the specific electrical resistance (SER) was measured using a four-point probe array.

### CT and MRI compatibility

Conventional Ag/AgCl and MWCNT electrodes were attached to the scalp, and CT (Toshiba, X vision) and low (Hitachi, AIRIS Vento 0.3 T) and high (Toshiba, Advantage Titan, 1.5 T) MRI scans were performed using commercially available equipment. Two conventional Ag/AgCl electrodes were placed on the scalp in two volunteers in low magnetic field MRI. Artifacts on the images generated from the EEG electrodes were examined by senior author R.K. In high magnetic field MRI, metal electrodes are prohibited for safety reasons. CT scanning was conducted under standard conditions, and MRI sequences recommended by the manufacturers were used. T1-, T2-, and diffusion-weighted images (DWI) as well as fluid-attenuated inversion recovery (FLAIR) images were obtained, and MR angiography was conducted.

### Evoked potentials

Ordinary metal and MWCNT electrodes were attached to the same area using commercially available medical conductive paste (Elefix Z-401CE, Nihon Kohden, Tokyo, Japan) and connected to electrophysiological monitoring equipment (Neuropack MEB-2200, Nihon Kohden, Tokyo, Japan). The contact impedance of the electrodes attached to the scalp was measured, followed by measurement of somatosensory-evoked potentials (SEPs), auditory brainstem responses (ABRs), and visually evoked potentials (VEPs). In the VEP and SEP tests, stimuli were applied 100 times and the mean potentials were calculated. In the ABR test, stimuli were applied 1,000 times. In the analysis of VEPs, we defined peak positive waves at around 100 msec as P100, and positive waves preceding and following P100 as N1 and N2, respectively. The amplitude between P100 and N2 was calculated [11].

In the SEP experiment, the amplitudes of N20 and P25 were calculated [12]. The analysis of ABRs involved identification of the amplitudes of five waves. The amplitude of an ABR wave was defined as the height of the V wave from the baseline [13]. In order to confirm reproducibility, ten volunteers underwent all of the evoked potential experiments three times each. In the first part of the experiments, the fabricated electrodes were checked in a shielded room. Then the experiments were conducted in an operating room, with intraoperative CT scanning equipment [1, 3], and the ICU of the University of Fukui Hospital — almost the same clinical environment in which medical equipment often generates AC-power noise. The mechanical robustness and electrical stability of the equipment were evaluated. In the present study, the skin conditions of the participants were checked. Acute inflammation of the skin was checked for just after each experiment. Chronic scalp changes and irritation were investigated 1 week later. A total of 60 MWCNT electrodes were placed on the skin, and the obtained data were analyzed by the Mann–Whitney U-test using computer software.

## Results

### Specifications of multi-walled carbon nanotube electrodes

**Figure 2:**
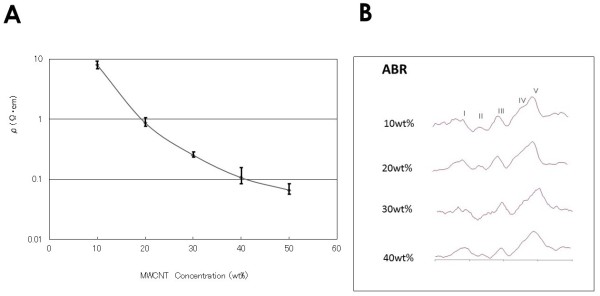
**A: The correlation between the specific electrical resistance (SER) and MWCNT concentration.** (N = 5 each). **B**: ABRs at different MWCNT concentrations. All of the ABRs have distinguishable peaks in the shielded room.

The newly developed electrode is very thin, with a thickness of 250 μm, and the form resembles a sheet of paper (Figure 1). The actual weight was around 25 mg each. The higher the MWCNT concentration, the lower the SER (Figure 2A). Even when the MWCNT concentration was 50%, the SER was approximately 0.061 ± 0.016 Ω/cm. The SER of the newly developed electrode was higher than we had expected and above that of ordinary metal electrodes, measured as 1.18 × 10^-5^ Ω/cm. Electrodes with an MWCNT concentration of 50% or higher were brittle and easily damaged. The SEPs, VEPs, and ABRs of electrodes with varying concentrations were assessed in a shielded room. The evoked potential waveforms were presumably not correlated with the concentrations of the MWCNT electrodes (Figure 2B). We decided to use MWCNT electrodes with a concentration of 20% in the subsequent experiments done in the operating room and the ICU, because they were soft and fitted the curve of the head very well.

### CT and MRI compatibilities

**Figure 3:**
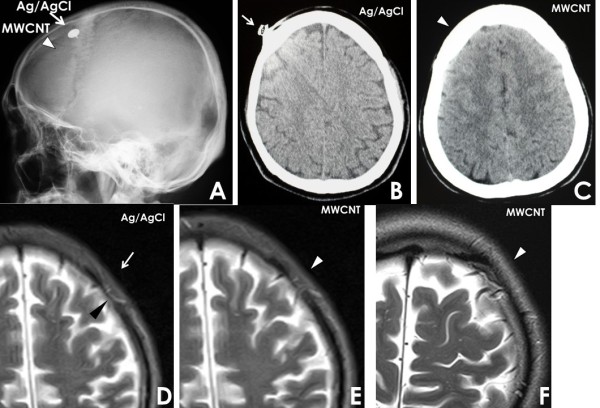
**A: X-ray images, radiolucent MWCNT (triangle) and Ag/AgCl (arrow) electrodes. B**: CT scan image revealing radiating artifacts from silver electrodes (arrow). **C**: No artifact from MWCNT electrode (triangle). **D**: Ag/AgCl electrode (arrow) producing linear susceptibility artifact (black triangle) in subcutaneous tissue in T2 WI on 0.3 T MRI. **E**: no artifact from MWCNT electrode (triangle) in 0.3 T MRI. **F**: no artifact from MWCNT electrode (triangle) in high magnetic field 1.5 T MRI.

Ten volunteers with electrodes underwent image scanning: MRI (2 cases) and CT (8 cases). The X-ray images clearly showed that conventional silver electrodes are metallic and that MWCNT electrodes (present next to the conventional ones) are radiolucent (Figure 3A). Linear artifacts were seen in a CT image involving a silver electrode, presumably because of metals contained in the electrode (Figure 3B). It was difficult to identify the brain tissues beneath the artifacts. On an axial CT image, MWCNT electrodes were not seen and there was no artifact in the skull (Figure 3C). Moderate-level magnetic artifacts and linear ones under subcutaneous tissues were identified on low (0.3 T) MR images (Figure 3D). There were no artifacts on either high or low MR images involving MWCNT electrodes (Figure 3E and F). All of the obtained image sequences, including T1, T2, DW, FLAIR, and MRA images, provided high-quality anatomical images without artifacts that might cause clinical problems. There were no signs or symptoms posing risks during examinations. After the imaging experiments, the electrodes were used in electrophysical experiments in the operating room and the ICU. As the evoked potentials were obtained as usual, we judged that the MWCNT electrodes were stable and did not degenerate after multiple CT and MRI tests.

### Evoked potentials

**Figure 4:**
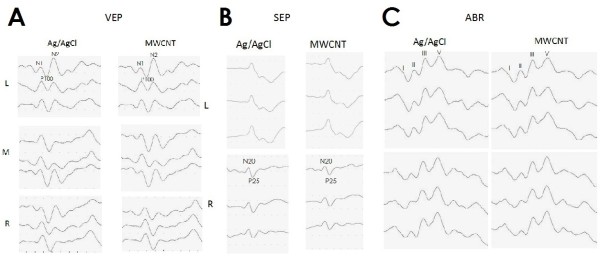
**Representative data of evoked potentials.** Electrodes placed parallel. **A**: VEP sampled simultaneously with Ag/AgCl (left) and MWCNT electrode (right) in a volunteer. L: left occipital, M middle occipital around confluence, R: right occipital. The quality and signal-to-noise ratio of data obtained with MWCNT electrodes were almost equal to what is obtained using conventional metal electrodes. **B**: SEP data by Ag/AgCl (left) and MWCNT electrode (right) in a volunteer. **C**: ABR samples by Ag/AgCl (left) and MWCNT electrode (right).

**Figure 5:**
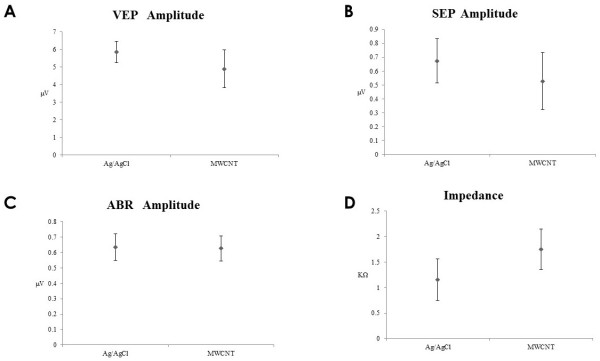
**Amplitude of each evoked potentials. A**; VEP, **B**; SEP, **C**; ABR. There was no significant difference between Ag/AgCl (left) and MWCNT electrode (right). N = 30 each. **D**: The impedance of Ag/AgCl (left) and MWCNT (right) electrodes. The impedance of metal electrodes was significantly lower (P < 0.05). The difference in the impedance between electrodes from the two companies was very small. The impedance was 5 kΩ or lower (N = 60 each).

The SEPs, ABRs, and VEPs were very stable in the ten volunteers with silver and MWCNT electrodes. There were no differences between the waveforms of recorded from MWCNT and silver electrodes (Figure 4). Also, no differences were noted in the amplitudes of the SEPs, ABRs, and VEPs (Figure 5 A–C). The impedance of the MWCNT electrodes was significantly higher than that of the silver electrodes (Figure 5D). However, none of the 60 experiments were discontinued just because the impedance was higher than 5 kΩ. Although approximately one hour was required to conduct an electrophysiological experiment involving one person, the initial impedance could be maintained throughout each experiment. There were no significant differences in the results of the experiments conducted in the ICU and the operating room.

### Skin complications

There was no damage or complication involving the skin, including skin eruptions, irritation, burns, bleeding, and infections, based on the use of the MWCNT electrodes.

## Discussion

At present, intraoperative electrophysiological monitoring for complicated clinical cases is essential [12, 14–16]. In addition to easy-to-use intraoperative imaging equipment, it is also important that EEG electrodes can be attached easily, safely, promptly, and in a cost-effective manner. A variety of electrodes have been developed, such as staple [6], spiral scalp [17], peg-screw [14], carbon-wire [7], and conductive plastic electrodes [5]. Considering that imaging equipment is used during surgery, we need to develop a new EEG electrode that is: markedly conductive with an impedance of less than 5 kΩ [18], permeable to X-rays and MRI, thin and not an obstacle in the surgical field, easy to attach to the scalp, disposable and inexpensive, and can be easily produced. The thin-film EEG electrode is made from carbon nanotubes, which are resistant to acid and high temperatures and significantly functional with unique electrical characteristics [8]. Carbon nanotubes are applicable in the fields of nanotechnology, electricity, and medicine, particularly for their marked mechanical strength and electrical characteristics [8, 9, 15]. Nanotubes are classified into single- and multi-walled. In theory, the MWCNT is a metal nanotube that can transmit an electrical current with a density of 4 × 10^9^ A/cm^2^, which is 1,000 times higher than the current density that can be transmitted by a copper tube [9]. The MWCNT fulfilled all the requirements for an electrode used for surgery. The evoked potentials of the MWCNT electrode were measured at 10%- to 40%-MWCNT dispersion concentration levels. The evoked potentials were similar to those of silver electrodes. Electrode impedance was modeled as a parallel circuit of a capacitor and a resistor. The SER was representative of a resister, not a capacitor. The MWCNT satisfied these requirements even when the concentration of the electrode was 10%, presumably because of its low impedance [10]. In fact, the impedance of 20% MWCNT electrodes between the electrode and the scalp was 1.7 KΩ. The capacitance might be large enough. Conductive wires and non-metal cables with a higher level of conductivity than that of copper ones can also be produced, as reported by recent studies [15]. Electrophysiological monitoring has increasingly been adopted and used, along with intraoperative imaging, CT, and MRI equipment [1, 3, 4] as well as cerebral angiography [2]. Medical devices with metals embedded are restricted from the viewpoint of radiology because CT and MRI may generate linear and magnetic susceptibility artifacts. According to a previous study, a silver-epoxy-coated, plastic conductive EEG electrode has been developed, which allows the measurement of brain waves without generating artifacts in CT. However, using this electrode in MRI may cause a magnetic field gradient between the electrode and skin, generating tumor-like artifacts on the image [5]. Metals are not allowed to be used in magnetic fields generated by MRI equipment in actual clinical settings [19]. However, the newly developed non-metal electrode can be used not only for intraoperative CT and MRI, but also in endovascular treatment and balloon occlusion tests.

As heavy electrodes easily detach from the skin during surgery, light electrodes should be used. The thin, non-metal electrode is desirable since it can be used for any type of neurosurgery. Although EGG electrodes are often contaminated with blood, water, and disinfectants, they are generally re-used. Electrodes used for patients with infectious diseases must be disposed of. The MWCNT is not very expensive; the price of one MWCNT electrode is less than five USD. However in Japan, the price of one needle electrode is approximately 20 USD, and one spiral-shaped electrode costs about 40 USD [6].

There has been serious concern over the toxicity of carbon nanotubes. In an experiment, MWCNT electrodes were attached to the lungs of rat and mouse models, and granulomas formed [20]. Furthermore, according to a previous study, a large volume (3 μg/mouse) of intraperitoneally injected MWCNT caused mesothelioma [21]. There have also been health concerns over the absorption of aerosolized MWCNT electrodes. To address these problems, carbon nanotubes used for the newly developed electrode are contained in polymethylmethacrylate (PMMA), a substance widely used for surgery and dental treatment [22], to prevent them from being aerosolized. Researchers and companies have been conducting studies on the safety of carbon nanotubes to promote application to a variety of products [21, 23].

## Conclusion

A very thin electrode was created using MWCNTs. This electrode allowed the measurement of brain waves and evoked potentials as accurately as conventional silver electrodes, without distorting X-ray, CT, and MR images. The MWCNT electrode is cost-effective and has great potential as surgical equipment.
